# Age-related variation in immunity in a wild mammal population

**DOI:** 10.1111/j.1474-9726.2011.00771.x

**Published:** 2012-02

**Authors:** Daniel H Nussey, Kathryn Watt, Jill G Pilkington, Rose Zamoyska, Tom N McNeilly

**Affiliations:** 1 Centre for Immunity, Infection and Evolution, School of Biological Sciences, University of EdinburghWest Mains Road, Edinburgh, UK; 2 Institute of Evolutionary Biology, School of Biological Sciences, University of EdinburghWest Mains Road, Edinburgh, UK; 3 Institute of Immunology and Infection Research, School of Biological Sciences, University of EdinburghWest Mains Road, Edinburgh, UK; 4 Moredun Research Institute, Pentlands Science ParkBush Loan, Midlothian, UK

**Keywords:** lymphocytes, immunosenescence, inflammation, naïve T cells, soay sheep, γδ T cells

## Abstract

Age-related changes in immunity are well documented in humans and laboratory mammals. Using blood samples collected from wild Soay sheep, we show that pronounced differences in T-cell subsets and inflammatory markers amongst age classes are also evident under natural conditions. These shifts parallel those observed in mammals experiencing protected environments. We found progressive declines in the proportion of naïve CD4 T cells with age, a precipitous drop in γδ T cells after the second year of life and an increase in acute phase protein levels amongst geriatric sheep. Our findings suggest immune aging patterns observed in laboratory and domestic mammals may generalize to more complex, challenging environments and could have fitness costs under natural conditions.

Research in humans and laboratory mammals has demonstrated profound changes in immunity with age, including declines in the ratio of naïve to memory T lymphocytes and increases in inflammatory markers (Linton & Dorshkind, 2004; Singh & Newman, 2011). Longitudinal studies suggest these changes may be important in age-related pathology and mortality in elderly humans and laboratory mice (Larbi *et al.*, 2008; Singh & Newman, 2011). However, the wider evolutionary significance of such age-related changes in mammals remains uncertain (Shanley *et al.*, 2009). We currently do not know whether immune aging patterns observed in the benign conditions experienced by modern humans and laboratory populations have any parallels in mammals experiencing parasite-rich, food-limited natural environments representative of those under which they actually evolved. Here, we present the first test for age-related differences in lymphocyte subsets and inflammatory markers in a wild mammal and report considerable similarity to the patterns observed in humans and laboratory mammals.

The population of Soay sheep (*Ovis aries*) in the Village Bay area of Hirta, St Kilda, has been closely monitored since 1985. It is unmanaged and unpredated with individuals experiencing food limitation over winter and challenges from micro- and macro-parasites (Clutton-Brock & Pemberton 2004). In August 2010, we collected blood samples from female lambs, yearlings, adults (2–6 years) and geriatrics (7–10 years) to examine age-related variation in immune measures known to change with age in humans or laboratory model systems (see Appendix S1).

T-cell populations were defined as helper (CD4+), naive (CD45RA+), regulatory (FoxP3+) or cytotoxic (CD8+), based on analogy with equivalent human and murine subpopulations. All measured T-cell subsets varied significantly amongst age classes, but we observed particularly notable declines in the proportion of naïve T helper cells and γδ T cells with age (Fig. 1). The proportion of T helper cells (CD4+) increased from around 25% of the total circulating lymphocyte population in lambs to 35% in geriatric sheep (*F*_3,43_ = 9.63, *P* < 0.001; Fig. 1A). Within this subset, the proportion of naïve helper T cells (CD4+ CD45RA+) declined progressively amongst age classes from around 35% to < 10% (*F*_3,42_ = 57.97, *P* < 0.001; Fig. 1B). Such a pattern is expected owing to declining thymic output of naïve T cells alongside their continuous antigenic activation and is consistent with findings in laboratory models and humans (Linton & Dorshkind, 2004), but has not previously been documented in a wild mammal to our knowledge. The proportion of regulatory T helper cells (CD4+ FoxP3+;’Tregs’), particularly those with a naïve phenotype (CD4+ FoxP3+ CD45RA+), declined with age (*F*_3,43_ = 12.19 and 18.15, respectively, both *P* < 0.001; Fig. 1C,D). The change in Tregs is in the opposite direction of that generally observed in mice and humans (Dejaco *et al.*, 2006), and it is not clear why this is the case. However, the decline in naïve Tregs is consistent with previous findings in humans (Booth *et al.*, 2010). The proportion of cytotoxic T cells (CD8+) was higher in geriatrics than other age classes but did not vary significantly between lambs and adults (*F*_3,43_ = 7.83, *P* < 0.001; Fig. 1E). Finally, the proportion of γδ T cells, which are known to circulate at high levels in young domestic ruminants (relative to humans and laboratory rodents) and decline with age in cattle (Hein & Mackay, 1991), decreased precipitously from around 20% in lambs and yearlings to < 5% in geriatric females (*F*_3,43_ = 32.22, *P* < 0.001; Fig. 1F).

**Fig. 1:**
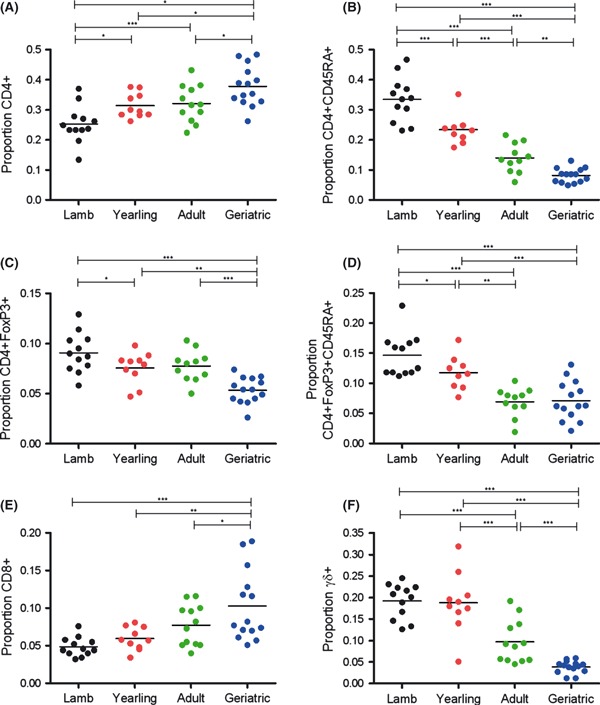
Age-related differences in T lymphocyte subsets in female sheep. Bars are mean values for each age class; lines above indicate significant post hoc tests comparing age groups (*P < 0.05; **P < 0.01; ***P < 0.001). Plots reflect the proportion of total lymphocyte population comprised of: (A) CD4+, (E) CD8+ and (F) γδ+ T cells; proportion of the CD4+ population comprised of (B) CD45RA+ naïve cells, (C) FoxP3+ regulatory cells; and (D) proportion of the CD4+ FoxP3+ cells that were CD45RA+ naïve cells.

Studies in humans frequently report increases in acute phase proteins and interleukin-6 (IL-6), a pro-inflammatory cytokine, in old age (Singh & Newman, 2011). We found that two acute phase proteins, haptoglobin and serum amyloid A, were higher on average in geriatric sheep than in yearlings and adults (*F*_3,42_ = 5.22 and *F*_3,40_ = 2.93, respectively, both *P* < 0.05; Fig. 2A,B). However, we did not find any age-related variation in IL-6 (*F*_3,43_ = 0.92, *P* = 0.44; Fig. 2C). IL-10, an anti-inflammatory cytokine, was significantly lower in lambs than in other age classes but did not vary from yearlings to geriatrics (*F*_3,43_ = 6.94, *P* < 0.001; Fig. 2D).

**Fig. 2:**
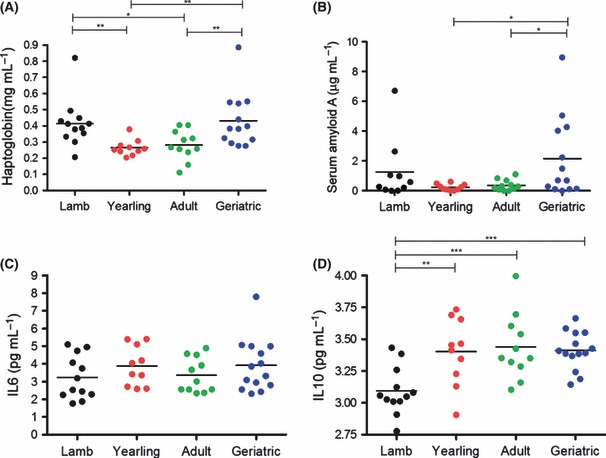
Age-related differences in (A) haptoglobin, (B) serum amyloid A, (C) interleukin-6 and (D) interleukin-10 in plasma from female sheep. Bars indicate mean values for each age class; lines above indicate significant *post hoc* tests comparing age groups (*P < 0.05; **P < 0.01; ***P < 0.001).

Our data complement accumulating evidence that declines in survival and reproduction with age are readily observable in the wild (Brunet-Rossinni & Austad, 2006) and that immune responses to antigenic challenge decrease with age in wild birds (Palacios *et al.*, 2011). Studies testing evolutionary predictions in natural populations can provide important insights into the origins and maintenance of genetic variation underlying immunity (e.g. Graham *et al.*, 2010; Räberg & Stjernman, 2003). Our relatively small, cross-sectional sample precluded us from detecting evolutionary trade-offs between growth and reproductive effort and our immune measures (see Appendix S2, Table S1). However, in providing the first evidence for age-related differences in T-cell subsets and acute phase proteins in a mammal experiencing ecologically realistic conditions, our data do suggest an important new degree of generality for patterns observed in the laboratory by immunologists. They also suggest that such age-dependent differences in immunity are targets for natural selection in wild mammals and highlight the potential for longitudinal research in wild animals to illuminate the evolutionary causes and consequences of variation in immunosenescence.

## Supporting information

**Appendix S1** Experimental Procedures.

**Appendix S2** Associations between immune measures and parasite burden, growth and reproduction.

**Table S1** Associations between immune measures and parasite burden, growth and reproduction.

As a service to our authors and readers, this journal provides supporting information supplied by the authors. Such materials are peer-reviewed and may be re-organized for online delivery, but are not copy-edited or typeset. Technical support issues arising from supporting information (other than missing files) should be addressed to the authors.
