# Supplementary material for: Age-related variation in immunity in a wild mammal population
Source: Aging Cell. 2012 Feb;11(1):178–80. doi: 10.1111/j.1474-9726.2011.00771.x (PMC3397677; doi:10.1111/j.1474-9726.2011.00771.x)
Supplement: Supplementary file 1 [file acel0011-0178-SD1.doc]

**Supporting information for**

**“Age-related variation in immunity in a wild mammal population”**

By Daniel H. Nussey, Kathryn Watt, Jill G. Pilkington, Rose Zamoyska, and Tom N. McNeilly

**Experimental Procedures**

***The study population & sample collection***

Soay sheep are descendants of domestic sheep that were present throughout northwest Europe during the Bronze Age and probably reached the island of Soay on the St Kilda archipelago 3000–4000 years ago. The study population live on the largest island of the archipelago, Hirta (57°49' N 8°34' W) from which humans and their modern domestic stock were evacuated in 1930. In 1932, around a hundred Soay sheep were introduced to Hirta from Soay and have since remained as an unmanaged population. Although they originate from an extremely primitive domestic stock, it is important to note that Soay sheep have been living in an entirely unmanaged state on St Kilda for likely more than three millennia. They experience fluctuating and often high levels of nutritional stress as well as infection with diverse parasites and pathogens, and have experienced at least several hundreds of generations of selection under such natural conditions. The genetic variation exhibited by and environmental conditions experienced by these animals therefore have the potential to be far removed from mammals in domestic, agricultural or laboratory conditions.

The population in the Village Bay area of Hirta has been subject to individual-based study since 1985 (Clutton-Brock & Pemberton 2004). Individuals are caught as newborn lambs, tagged, and subsequently monitored closely throughout their lifetimes. Every August, sheep from the study population are rounded up in a series of temporary traps, and are caught and measured with faecal and blood samples taken as part of the ongoing long-term study (Clutton-Brock & Pemberton 2004). On 10^th^ August 2010, we took additional blood samples from 47 sheep out of the total 210 sheep captured that day. Female sheep belonging to different major age classes were selected. Age classes were defined based on previous studies of variation in demographic rates (Catchpole *et al.* 2000; Coulson *et al.* 2001; Wilson *et al.* 2007): lambs (around 4 months old, N = 12), yearlings (around 16 months old, N = 10), adults (2-6 years old, N = 11) and geriatrics (≥7 years old, N = 14). In 2010 summer censuses of the population, these age classes represented 23.9, 14.1, 39.0, and 23.0 % (respectively) of the total number of tagged females observed in the study area. Previous studies have demonstrated marked declines in survival probability and reproductive performance and increases in parasite burden among female sheep after six years of age (Catchpole *et al.* 2000; Wilson *et al.* 2007; Hayward *et al.* 2009).

Blood samples were collected in Lithium heparin tubes. Plasma was prepared for analysis by centrifuging whole blood at 3000 rpm for 10 minutes and drawing off the upper plasma layer and storing it at -20°C. Blood leukocytes were purified and fixed at the field site for subsequent flow cytometry analysis by adding 1ml of whole blood to 5ml of warm (37°C) ammonium chloride lysing solution (1.5 M NH_4_Cl, 100 mM NaHCO_3_, 10 mM Na_2_EDTA), mixing gently and then centrifuging at 1200 rpm for 10 minutes. The cell pellet was washed once in 8 ml of cold phosphate buffered saline (PBS) then re-suspended in 2.5 ml 1 % paraformaldehyde in PBS and incubated at room temperature (RT) for 10 minutes. After a final wash in PBS, cells were re-suspended in 1 ml PBS containing 0.02 % NaN_3_. Samples were stored at 4°C prior to flow cytometry analysis. On return to Edinburgh, the number of fixed cells per sample was counted using an Innovatis CASY® cell counter. Results of analyses based on absolute cell numbers derived from these total counts and percentages from flow cytometry were broadly similar to those reported for proportions of different T cell types, and so only proportional data are discussed further here.

***Flow cytometry***

50 µl of fixed leukocyte suspensions were transferred to a round-bottomed 96 well plate, washed once in fluorescence activated cell sorting (FACS) buffer (5 % foetal calf serum, 0.05% sodium azide in PBS) and incubated in FACS buffer containing 20 % normal goat serum for 30 minutes at 4^o^C. For single-colour staining, cells were incubated with anti-ovine monoclonal antibodies (mAb) to CD4 (clone 17D, mouse IgG1, Mackay *et al.* 1988), CD8 (clone 72C, mouse IgG2a, Young *et al.* 1997), γδ TcR (clone 86D, mouse IgG1, Mackay *et al.* 1989) or the appropriate isotype control antibodies (mouse IgG2a anti-border disease virus (BDV) p125 ⁄ p80, clone VPM20, and mouse IgG1 anti-BDV p125 ⁄ p80, clone VPM21, Entrican *et al.* 1995) for 30 minutes at 4^o^C. After two washes in FACS buffer, cells were incubated with goat anti-mouse IgG [H+L] conjugated to Alexa Fluor® 647 (Invitrogen, Carlsbad, CA) for 30 minutes at 4^o^C. After final washing, cells were fixed in 1 % PFA in PBS for 10 minutes at RT before analysis on a BD FACSArray™ Bioanalyser (BD Biosciences, San Jose, CA) using the manufacturer’s acquisition software. Analysis of single-colour staining data was performed using FlowJo version 7.6.1 analysis software (TreeStar, San Carlos, CA).

For three colour staining of leukocytes for CD4, CD45RA and Foxp3, cells were incubated with both anti-ovine CD4 mAb conjugated to FITC (clone 44.38, mouse IgG2a, AbDserotec, Kidlington, UK) and anti-CD45RA (clone 73B, mouse IgG1, Mackay *et al.* 1990), or the appropriate isotype control antibodies (mouse IgG2a-FITC, eBioscience and mouse IgG1, clone VPM21) for 30 minutes at 4^o^C. Following washing in FACS buffer, cells were incubated with goat anti-mouse IgG1 conjugated to R-phycoerythrin (AbDserotec) for 30 minutes at 4^o^C, washed and then fixed in 1 % PFA in PBS for 10 minutes at RT. Cells were subsequently re-suspended in permeabilisation buffer consisting of 0.2 % saponin (Sigma-Aldrich, St. Louis, MO) diluted in FACS buffer containing 20 % normal rat serum and incubated overnight at 4^o^C. The following day cells were pelleted, and re-suspended in permeabilisation buffer without rat serum containing anti- mouse/rat Foxp3 mAb conjugated to Alexa Fluor® 647 (clone FJK-16s, rat IgG2a, eBioscience, San Diego, CA), which has previously been shown to cross-react with ovine FoxP3 (Rocchi *et al.* 2011), or an equivalent concentration of rat IgG2a-Alexa Fluor® 647 isotype control (eBioscience) and incubated for 1 hr at 4^o^C. After final washing, cells were again fixed in 1 % PFA in PBS for 10 minutes before analysis on a Cyan™ ADP flow cytometer (Beckman Coulter Inc., Fullerton, CA, USA) using the manufacturer’s acquisition and analysis software (Summit version 4.3).

***Acute phase protein and cytokine assays***

Haptoglobin and serum amyloid A have been established as major acute phase proteins in ruminants (Murata *et al.* 2004; Eckersall *et al.* 2007). We assayed plasma haptoglobin (Hp) concentration using a calorimetric kit (Phase™ Range Haptoglobin kit, Tridelta Development Ltd, Maynooth, Ireland; Catalogue number: TP801), followed the manufacturer’s instructions. Samples were assayed in duplicate and optical density values (ODs) were averaged for each sample before concentrations were calculated using the supplied standards. We assayed serum amyloid A (SAA) using a sandwich ELISA kit (Phase™ Range multispecies SAA ELISA kit, Tridelta Development Ltd; Catalogue number: TP802), following the manufacturer’s instructions. We diluted plasma samples to 1 in 50 in kit diluent prior to assays and ran all samples in duplicate, taking average ODs for each sample before concentrations were calculated using the supplied standards. Note that one geriatric female had extremely high ODs on both assays and two lambs had very high ODs for serum amyloid A, which were beyond the upper limit of the standard curve provided with the kits. Rather than extrapolating beyond the standards supplied, we excluded these individuals from further analysis. Plasma concentrations of IL-10 and IL-6 were assayed using ovine ELISA kits following manufacturer’s instructions (Sheep Interleukin-10 / Interleukin-6 ELISA kit, Cusabio Biotech Co., Catalogue numbers: CSB-E12817sh and CSB-E10116sh), using 50ul of plasma. Optical densities for all of the above assays were read using a Multiskan GO spectrophotometer (Thermo Fisher Scientific Inc.).

***Statistical analysis***

We tested for differences between age groups for each immune variable measured with one-way ANOVAs. *Post hoc* t tests were used to identify which specific contrasts between groups were significant. Note that while several of the immune measures were not perfectly normally distributed, most were approximately normal and conducting analysis using standard transformations (arcsine square root transformations for proportions, log transformations for optical densities or concentrations) did not meaningfully alter our results (data not shown). All analyses were conducted in R version 2.11 (R Core Development Team 2005).

***References for experimental procedures***

Catchpole EA, Morgan BJT, Coulson TN, Freeman SN, Albon SD (2000). Factors influencing soay sheep survival. *J. R. Stat. Soc. Ser. C - Appl. Stat.* **49**, 453-472.

Clutton-Brock TH, Pemberton JM (2004). *Soay sheep: Dynamics and selection in an island population*. Cambridge: Cambridge University Press.

Coulson T, Catchpole EA, Albon SD, Morgan BJT, Pemberton JM, Clutton-Brock TH, Crawley MJ, Grenfell BT (2001). Age, sex, density, winter weather, and population crashes in soay sheep. *Science*. **292**, 1528-1531.

Eckersall PD, Lawson FP, Bence L, Waterston MW, Lang TL, Donachie W, Fontaine MC (2007). Acute phase protein response in an experimental model of ovine *Caseous lymphadenitis*. *BMC Vet. Res*. **3**, 35.

Entrican G, Dand A , Nettleton PF (1995). A double monoclonal-antibody ELISA for detecting pestivirus antigen in the blood of viremic cattle and sheep. *Vet. Microbiol*. **43**, 65-74.

Hayward AD, Wilson AJ, Pilkington JG, Pemberton JM, Kruuk LEB (2009). Ageing in a variable habitat: Environmental stress affects senescence in parasite resistance in st kilda soay sheep. *Proc. R. Soc. B*. **276**, 3477-3485.

Mackay CR, Beya MF, Matzinger P (1989). Gamma-delta T-cells express a unique surface-molecule appearing late during thymic development. *Europ. J. Immunol*. **19**, 1477-1483.

Mackay CR, Hein WR, Brown MH , Matzinger P (1988). Unusual expression of CD2 in sheep - implications for T-cell interactions. *Europ. J. Immunol*. **18**, 1681-1688.

Mackay CR, Marston WL, Dudler L (1990). Naive and memory T-cells show distinct pathways of lymphocyte recirculation. *J. Exp. Med.* **171**, 801-817.

Murata H, Shimada M, Yoshioka M (2004). Current research on acute phase proteins in veterinary diagnosis: an overview. *Vet. J*. **168**, 28-40.

R Core Development Team (2005) R: A language and environment for statistical computing. . R Foundation for Statistical Computing, Vienna, Austria. [http://www.R-project.org](http://www.R-project.org/).

Rocchi MS, Wattegedera SR, Frew D, Entrican G, Huntley JF, McNeilly TN (2011). Identification of CD4+CD25high Foxp3+ T cells in ovine peripheral blood. *Vet. Immunol. Immunopathol*. doi:10.1016/j.vetimm.2011.07.010.

Wilson AJ, Nussey DH, Pemberton JM, Pilkington JG, Donald A, Pelletier F, Clutton-Brock TH, Kruuk LEB (2007). Evidence for a genetic basis of aging in two wild vertebrate populations. *Curr. Biol.* **17**, 2136-2142.

Young AJ, Marston WL, Dessing M, Dudler L , Hein WR (1997). Distinct recirculating and non-recirculating B-lymphocyte pools in the peripheral blood are defined by coordinated expression of CD21 and L-selectin. *Blood*. **90**, 4865-4875.

**Appendix A: Associations between immune measures and parasite burden, growth and reproduction**

Evolutionary theory predicts that trade-offs between different fitness functions, such as growth, reproduction and physiological maintenance, constrain the action of natural selection on life histories and can maintain genetic variation underlying phenotypes (Roff 1992; Stearns 1992). Immunity is considered a central component of the maintenance axis of such trade-offs, as it is crucial for survival in parasite-filled natural environments (Sheldon & Verhulst 1996; Schmid-Hempel 2011). Theory also predicts that early reproduction is traded off against longer term somatic maintenance, such that increased early-life reproduction is expected to come at a cost of earlier and more rapid senescence (Williams 1957; Kirkwood & Rose 1991). Recent work on wild ungulate populations provides support for the existence of reproductive costs of immunity and that early-life reproduction exacerbates reproductive ageing rates (Nussey *et al.* 2006; Graham *et al.* 2010). Despite growing interest in age-related variation in immunity in wild vertebrates and some emerging evidence for cross-sectional declines in swelling or antibody responses to a challenge in wild birds (Palacios *et al.* 2011), tests of the prediction that growth, infection and reproduction in earlier life should exacerbate immunosenescence are lacking as is an appreciation of the real fitness consequences of immunosenescence itself. A major challenge remains to begin to measure the same immunological variables in wild animals as those at the current focus of in research into aging in model organisms. The main text of our work reports the first data to make this step, and suggests potential similarities that should pave the way for follow-up research to begin to address the wider evolutionary causes and consequences of immunosenescence in natural populations.

We selected a small, age structured, cross-sectional sample of animals with the expressed intention of examining age-dependent variation across a broad suite of immune markers relevant to immunosenescence, based on research from humans and model laboratory organisms. Since the data was collected as part of a long-term, individual-based study, there was accompanying data on parasite burden (faecal strongyle nematode egg counts), growth rates since birth in lambs and reproductive data in adults. While this does make tests of predicted associations between immune measures, parasite burdens, growth and reproduction possible it should be noted this was not the purpose of this particular study. Furthermore, documenting significant relationships of this sort in natural populations, where a host of confounding variables and sources of heterogeneity typically need to be accounted for before trade-offs can be revealed, demands large sample sizes and longitudinal data (van Noordwijk & de Jong 1986; van de Pol & Wright 2009). Furthermore, since growth, condition, reproductive performance, parasite burden and – as our current data suggests – immunological variables all vary with age in this population, relatively large samples within each age class would be required to identify significant trade-offs or associations over and above the changes observed with age.

Nonetheless, for completeness, we have run linear models of each of our 10 immunological measures (Figures 1 and 2) to test for any evident associations with parasite burden, growth and reproduction. Results are given in Table S1. We assessed associations with strongyle nematode faecal egg counts (see Craig *et al.* 2006 for measurement details) in models in which age class was also included as a factor, to account for age differences. We then assessed associations between growth rates (weight in August minus birth weight corrected for measurement age) and immunological measures in the 12 lambs with linear regression. Finally we tested for associations between immunological measures and whether or not a female reproduced in the spring preceding measurement using linear models. Although Soay sheep ewes can breed in their first year, only 2 of our 10 measured yearlings did so and therefore we restricted this latter analysis to adults and geriatrics (of whom 7 out of 25 failed to breed that year). We fitted whether or not a female bred as a two-level factor along with age class (adult or geriatric) into models of each immunological variable.

Our results, presented in Table S1, reveal very little evidence for associations over and above those associated with age-related differences. This is not surprising given the small (10-14) sample sizes available for each age class, nor should it be taken as any kind of evidence against predicted trade-offs with growth or reproduction. The results simply serve to illustrate the rather considerable challenges associated with understanding the evolutionary causes and consequences of variation in ageing patterns in natural populations (Monaghan *et al.* 2008; Nussey *et al.* 2008). The observed patterns of age-related variation in diverse immune measures, and their apparent consistency with qualitative patterns in model organisms (Figures 1 and 2) should serve as impetus for larger scale, follow-up studies in wild vertebrates which focus on trying to measure key immunological markers longitudinally across the lifetimes of individuals and relate these to growth, infection and reproduction in earlier life. Combined with analyses focussed on understanding the fitness consequences of immunological variation in later adulthood in natural populations, such research will help determine the evolutionary causes and consequences in variation in the complex process of immunosenescence.

***References for Appendix A***

Craig BH, Pilkington JG , Pemberton JM (2006). Gastrointestinal nematode species burdens and host mortality in a feral sheep population. *Parasitology*. **133**, 485-496.

Graham AL, Hayward AD, Watt KA, Pilkington JG, Pemberton JM , Nussey DH (2010). Fitness correlates of heritable variation in antibody responsiveness in a wild mammal. *Science*. **330**, 662-665.

Kirkwood TBL , Rose MR (1991). Evolution of senescence: Late survival sacrificed for reproduction. *Philos. Trans. R. Soc. B*. **332**, 15-24.

Monaghan P, Charmantier A, Nussey DH , Ricklefs RE (2008). The evolutionary ecology of senescence. *Funct. Ecol.* **22**, 371-378.

Nussey DH, Coulson T, Festa-Bianchet M , Gaillard JM (2008). Measuring senescence in wild animal populations: Towards a longitudinal approach. *Funct. Ecol.* **22**, 393-406.

Nussey DH, Kruuk LEB, Donald A, Fowlie M , Clutton-Brock TH (2006). The rate of senescence in maternal performance increases with early-life fecundity in red deer. *Ecol. Lett.* **9**, 1342-1350.

Palacios MG, Winkler DW, Klasing KC, Hasselquist D , Vleck CM (2011). Consequences of immune system aging in nature: A study of immunosenescence costs in free-living tree swallows. *Ecology*. **92**, 952-966.

Roff DA (1992). *The evolution of life histories: Theory and analysis.* London: Chapman & Hall.

Schmid-Hempel P (2011). *Evolutionary parasitology: The integrated study of infections, immunology, ecology and genetics*. Oxford: Oxford University Press.

Sheldon BC , Verhulst S (1996). Ecological immunology: Costly parasite defences and trade-offs in evolutionary ecology. *Trends Ecol. Evol.* **11**, 317-321.

Stearns SC (1992). *The evolution of life histories*. Oxford: Oxford University Press.

van de Pol MV , Wright J (2009). A simple method for distinguishing within- versus between-subject effects using mixed models. *Anim. Behav.* **77**, 753-758.

van Noordwijk AJ , de Jong G (1986). Acquisition and allocation of resources - their influence on variation in life-history tactics. *Am. Nat.* **128**, 137-142.

Williams GC (1957). Pleiotropy, natural selection and the evolution of senescence. *Evolution*. **11**, 398-411.

**Table S1. Associations between immune measures and parasite burden, growth and reproduction**

Table listing results of linear models testing the association between immunological variables and strongyle nematode faecal egg counts (models include all individuals and control for differences among age groups), lamb growth rates (models include only data from 12 lambs), and reproduction (whether or not a female bred the preceding spring, including only adults and geriatrics with differences among those two age classes controlled for). The direction of the effect, standard errors and significance are reported in each case.

| Immune measure | Faecal egg count | Growth | Reproduction* |
| --- | --- | --- | --- |
| CD4+ | b = 0.007 (± 0.005 SE)  F_(1,38)_ = 2.30, P = 0.14 | b = -1.33 (± 1.00 SE)  F_(1,10)_ = 1.75, P = 0.22 | b = 1.05 (± 2.98 SE)  F_(1,22)_ = 0.12, P = 0.73 |
| CD8+ | b = -0.001 (± 0.002 SE)  F_(1,38)_ = 0.33, P = 0.57 | b = -0.27 (± 0.21 SE)  F_(1,10)_ = 1.76, P = 0.21 | b = -3.23 (± 1.68 SE)  F_(1,22)_ = 3.69, P = 0.07 |
| γδ+ | b = -0.002 (± 0.003 SE)  F_(1,38)_ = 0.62, P = 0.43 | b = -0.98 (± 0.65 SE)  F_(1,10)_ = 2.27, P = 0.16 | b = 0.70 (± 1.69 SE)  F_(1,22)_ = 0.18, P = 0.68 |
| CD4+CD45RA+ | b = 0.003 (± 0.004 SE)  F_(1,37)_ = 0.55, P = 0.46 | b = -0.53 (± 1.31 SE)  F_(1,10)_ = 0.16, P = 0.69 | b = -0.50 (± 1.69 SE)  F_(1,22)_ = 0.09, P = 0.77 |
| CD4+FoxP3+ | b = -0.000 (± 0.001 SE)  F_(1,38)_ = 0.04, P = 0.84 | b = -0.09 (± 0.35 SE)  F_(1,10)_ = 0.06, P = 0.81 | b = -0.16 (± 0.65 SE)  F_(1,22)_ = 0.06, P = 0.80 |
| CD4+FoxP3+CD45RA+ | b = 0.003 (± 0.002 SE)  F_(1,37)_ = 2.04, P = 0.16 | b = -0.07 (± 0.63 SE)  F_(1,10)_ = 0.01, P = 0.91 | b = -1.75 (± 1.30 SE)  F_(1,22)_ = 1.82, P = 0.19 |
| Haptoglobin | b = 0.000 (± 0.001 SE)  F_(1,37)_ = 0.05, P = 0.82 | b = 0.04 (± 0.02 SE)  F_(1,10)_ = 2.18, P = 0.17 | b = 0.14 (± 0.23 SE)  F_(1,22)_ = 0.37, P = 0.55 |
| Serum amyloid A | b = -0.002 (± 0.001 SE)  F_(1,35)_ = 2.10, P = 0.16 | b = 0.09 (± 0.39 SE)  F_(1,10)_ = 0.05, P = 0.83 | b = 1.27 (± 2.03 SE)  F_(1,22)_ = 0.39, P = 0.54 |
| IL-6 | b = 0.001 (± 0.001 SE)  F_(1,38)_ = 0.68, P = 0.42 | b = 0.30 (± 0.20 SE)  F_(1,10)_ = 2.33, P = 0.16 | b = 0.40 (± 0.60 SE)  F_(1,22)_ = 0.46, P = 0.51 |
| IL-10 | b = 0.000 (± 0.001 SE)  F_(1,38)_ = 0.68, P = 0.42 | b = -0.02 (± 0.02 SE)  F_(1,10)_ = 0.75, P = 0.41 | b = -0.04 (± 0.04 SE)  F_(1,22)_ = 1.33, P = 0.26 |

* for reproduction, b reflects the estimated difference between means of breeders minus non-breeders
